# Heme oxygenase-1 prevents non-alcoholic steatohepatitis through suppressing hepatocyte apoptosis in mice

**DOI:** 10.1186/1476-511X-9-124

**Published:** 2010-10-28

**Authors:** YueMin Nan, RongQi Wang, SuXian Zhao, Fang Han, Wen Juan Wu, LingBo Kong, Na Fu, Li Kong, Jun Yu

**Affiliations:** 1 Department of Traditional and Western Medical Hepatology, Third Hospital of Hebei Medical University, Shijiazhuang, PR China; 2 Institute of Digestive Disease and Department of Medicine and Therapeutics, The Chinese University of Hong Kong, Hong Kong

## Abstract

**Objective:**

Heme oxygenase-1 (HO-1), the rate-limiting enzyme in heme catabolism, has been reported to have potential antioxidant properties. However, the role of HO-1 on hepatocyte apoptosis remains unclear. We aim to elucidate the effects of HO-1 on oxidative stress related hepatocellular apoptosis in nutritional steatohepatitis in mice.

**Methods:**

C57BL/6J mice were fed with methionine-choline deficient (MCD) diet for four weeks to induce hepatic steatohepatitis. HO-1 chemical inducer (hemin), HO-1 chemical inhibitor zinc protoporphyrin IX (ZnPP-IX) and/or adenovirus carrying HO-1 gene (Ad-HO-1) were administered to mice, respectively. Hepatocyte apoptosis was evaluated by terminal deoxynucleotidyl transferase dUTP nick-end labeling (TUNEL) assay, the mRNA and protein expression of apoptosis related genes were assayed by quantitative real-time PCR and Western blot.

**Results:**

Hepatocyte signs of oxidative related apoptotic injury were presented in mice fed with MCD diet for 4 weeks. Induction of HO-1 by hemin or Ad-HO-1 significantly attenuated the severity of liver histology, which was associated with decreased hepatic lipid peroxidation content, reduced number of apoptotic cells by TUNEL staining, down-regulated expression of pro-apoptosis related genes including Fas/FasL, Bax, caspase-3 and caspase-9, reduced expression of cytochrome p4502E1 (CYP2E1), inhibited cytochrome c (Cyt-c) release, and up-regulated expression of anti-apoptosis gene Bcl-2. Whereas, inhibition of HO-1 by ZnPP-IX caused oxidative stress related hepatic injury, which concomitant with increased number of TUNEL positive cells and up-regulated expression of pro-apoptosis related genes.

**Conclusions:**

The present study provided evidences for the protective role of HO-1 in preventing nutritional steatohepatitis through suppressing hepatocyte apoptosis in mice.

## Introduction

Non-alcoholic steatohepatitis (NASH) is a chronic progressive liver disease which comprises steatosis, balloon degeneration, inflammation, and fibrosis in varying degrees [1]. The estimated prevalence of NASH is 3%-5% in general population [2]. Once NASH occurs, about 30% ~ 50% of individuals demonstrate advanced fibrosis or cirrhosis within a decade [3]. Up to now, the pathogenesis of NASH leading to disease progression remains poorly understood. The most widely accepted explanation is the two hit hypotheses [4], in which hepatocellular apoptotic response associated with oxidative stress is considered to be the critical "hit" [5-8] in the transition from benign steatosis to steatohepatitis.

Heme oxygenase-1 (HO-1) is a stress-responsive protein induced by various oxidative agents, and plays a fundamental role against the oxidative process [9]. It cleaves pro-oxidant heme into equimolar amounts of carbon monoxide (CO), biliverdin/bilirubin (BV/BR), and free iron [10]. These enzymatic reaction products have significant and useful biological properties, such as anti-oxidant, anti-inflammatory and anti-apoptotic activities [11-14]. A lack of HO-1 in either transgenic mice or in humans significantly increases apoptotic cell death [15,16]. Although a role of HO-1 as an antioxidant has been reported in many studies, the therapeutic potential of HO-1 in steatohepatitis through mediating apoptosis is still unknown. In this study, we examine the effect of HO-1 on hepatocellular apoptosis in the pathogenesis of steatohepatitis in mice.

## Materials and methods

### Animals and treatments

Eight-week-old male C57BL/6J mice were bred and housed as previously described [17]. Mice were randomly divided into 7 groups (6 mice per group): 1) MCD group, mice fed methionine-choline deficient diet (ICN, Aurora, Ohio, USA); 2) control group, mice fed MCD diet supplemented with choline bitartate (2 g/kg) and DL-methionine (3 g/kg) (ICN, Aurora, Ohio); 3) MCD+hemin group, mice fed MCD diet administered with HO-1 chemical inducer hemin (30 μmol/kg) by intraperitoneal (i.p.) injections three times per week; 4) MCD+ZnPP group, mice fed MCD diet administered with HO-1 inhibitor ZnPP-IX (20 μmol/kg) by i.p. injections three times per week; 5) MCD+Ad-GFP group, mice fed control diet administered with adenovirus encoding green fluorescent protein (2.5 × 10^8 ^Plaque-forming units (pfu) by i.p. injections two times per week; 6) MCD+Ad-HO-1 group, mice fed MCD diet administered with, adenovirus encoding the full-length mouse HO-1 (2.5 × 10^8 ^pfu) (Ad-HO-1) by i.p. injections two times per week; 7) MCD+hemin+Ad-HO-1 group, mice fed MCD diet administered with hemin and Ad-HO-1. At the end of the experiment for 4 weeks, all of the animals were sacrificed after overnight fasting. Livers were weighed and fixed in 10% formalin for histological analysis or snap-frozen in liquid nitrogen followed by storage at -80°C freezer until required. All the protocols and procedures were carried out in accordance with the guidelines of the Hebei Committee for Care and Use of Laboratory Animals and were approved by the Animal Experimentation Ethics Committee of the Hebei Medical University.

### Construction of recombinant adenovirus

A recombinant adenovirus containing the entire coding sequence of mouse HO-1 (Ad-HO-1) and control adenovirus encoding green fluorescent protein (Ad-GFP) were purchased from Tianjin Saier Biochemistry company limited (Tianjin, China). Adenovirus was propagated, isolated in human embryonic kidney 293 (HEK 293) cells and purified with Adeno-X Virus Purification kit (Clontech, Mountain View, CA, USA). Titer of the viral solution was determined by Adeno-X Rapid Titer kit (Clontech). The virus was stored at -80°C until use. Mice were given intraperitoneal injection of Ad-HO-1 or Ad-GFP at an amount of 2.5 × 10^8 ^PFU suspended in 100 μl phosphate-buffered saline two times per week.

### TUNEL assay

4 μm liver sections were deparaffinized in xylene and hydrated in graded ethanol, and terminal deoxynucleotidyl transferase dUTP nick-end labeling (TUNEL) assay was performed following manufacturer's instructions and the apoptotic cells were identified using a Cell Death Detection kit (Roche Molecular Biochemicals, Mannheim, Germany). Ten random fields from 3 slides per group were examined, and the TUNEL-positive brown nuclei within the hepatocytes were counted as previously described [18]. The data were expressed as the number of TUNEL-positive cells/high- power field (× 400).

### Determination of thiobarbituric acid-reactant contents of the livers

Liver lipoperoxide levels in tissue-homogenate supernatants were estimated using the thiobarbituric acid-reactive substrances (TBARS) assay (Cell Biolabs, Inc. San Diego, CA) [19].

### Immunohistochemistry for HO-1

Immunostaining for HO-1 was performed in paraffin-embedded liver sections using the specific antibody (Santa Cruz Biotechnology, Santa Cruz, CA) and an avidin-biotin complex (ABC) immunoperoxidase method. Briefly, endogenous peroxidase activity was blocked by treating sections with 3% hydrogen peroxide. The primary polyclonal rabbit antibody anti-HO-1 (dilution 1:100) was applied and incubated overnight at 4°C. After extensive rinsing, the biotin-conjugated secondary antibody, ABC complex/horseradish peroxidase were applied for 30 minutes at room temperature. Peroxidase activity was visualized by applying diaminobenzidine to the sections, which were then counterstained with haematoxylin. Quantitative analysis of HO-1-stained liver sections was performed by morphometric analysis.

### Quantitation of hepatic messenger RNA expression levels

Total RNA was extracted from the frozen liver tissues by using RNA Trizol reagent (Invitrogen, Carlsbad, CA). Five microgram of total RNA for each sample was reverse transcribed into complementary DNA (cDNA), the cDNA was diluted 1/100 and 5 μl were used as a template per PCR reaction. The quantitative real-time PCR was performed on an ABI PRISM 7300 PCR System (Applied Biosystems, Foster City, CA) using Syber Green I GoTaq^® ^qPCR Master Mix (Promega BioSciences. Sunnyvale, CA). Expression levels of the target genes generated standard curves were normalized against an endogenous reference gene glyceraldehydes 3-phosphate dehydrogenase (GAPDH). For each sample and each gene, PCR were carried out in duplicate and repeated twice. The specific primer sequences were listed in Table 1.

**Table 1:** Primers for real-time quantitative PCR analysis.

Gene	Product length	Primer sequences
CYP2E1	199 bp	F 5'-AACAGAGACCACCAGCACA-3'
		R 5'-GGAAGGGACGAGGTTGATGA-3'
Fas	504 bp	F 5'-TGCGATTCTCCTGGCTGTGA-3'
		R 5'-GGTTCTGCGACATTCGGCTT-3'
FasL	345 bp	F 5'-GAGTTCACCAACCAAAGCCTT-3'
		R 5'-CAACCTCTTCTCCTCCATTAGC-3'
Bcl-2	100 bp	F 5'-GGATGACTTCTCTCGTCGCTAC-3'
		R 5'-TGACATCTCCCTGTTGACGCT-3'
Bax	239 bp	F 5'-GGTTGCCCTCTTCTACTTTGC-3'
		R 5'-TCTTCCAGATGGTGAGCGAG-3'
Cyt-C	157 bp	F 5'-CGGCTGCTGTGATTGTGAAT-3'
		F 5'-TGTCTTGTGTTTCCCGCCTT-3'
caspase-3	439 bp	F 5'-ACGCAGCCAACCTCAGAGA-3'
		R 5'-ATGAACCACGACCCGTCCT-3'
caspase-9	171 bp	F 5'-TCCTCTCTTCATCTCCTGCTTAG-3'
		R 5'-ACTACTCTCTGCTCCTTTGCTG-3'
GAPDH	450 bp	F 5'-ACCACAGTCCATGCCATCAC-3'
		R 5'-TCCACCACCCTGTTGCTG-3'

Abbreviations: CYP2E1, cytochrome p4502E1; Fas, death receptor; FasL, Fas Ligand; Cyt-C, cytochrome C; GAPDH, glyceraldehyde 3-phosphate dehydrogenase.

### Western blotting analysis of hepatic proteins

Total protein was extracted and concentration was measured by the Bradford method (DC protein assay; Bio-Rad, Hercules, CA). Equal amounts of protein (100 mg/well) were loaded onto 10% SDS-PAGE for each sample and proteins were transferred onto equilibrated polyvinylidene difluoride membranes (Amersham Biosciences, Buckinghamshire, UK) by electroblotting. Membranes were incubated overnight at 4°C with primary antibodies (Santa Cruz Biotechnology, Santa Cruz, CA). After incubation with the secondary antibody, proteins were detected by enhanced chemiluminescence (ECL, Amersham Corporation). The amount of protein expression was corrected by the amount of β-actin in the same sample and the bands were quantified by scanning densitometry using the digital Kodak Gel Logic 200 (Carestream Molecular Imaging, USA).

### Statistical analysis

All data are expressed as mean ± standard deviation (SD). Statistical analysis was carried out by one-way analysis of variance (ANOVA) and the Student-Newman- Keuls test for evaluating differences between groups using SPSS 13.0 (v.13.0; SPSS Inc., Chicago, III, USA). A *P*-value of less than 0.05 was considered statistically significant.

## Results

### Effect of HO-1 on hepatocyte apoptosis

**Figure 1:**
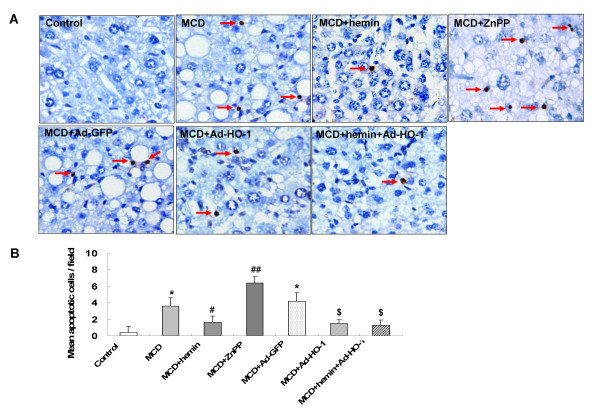
**Effect of HO-1 on hepatocyte apoptosis in mice fed with MCD diet at four weeks**. (A) TUNEL staining for hepatocyte apoptosis in liver sections from mice administrated: control diet; MCD diet; MCD diet treated with hemin, ZnPP-IX, Ad-GFP, Ad-HO-1 and combination of Ad-HO-1 and hemin, respectively. (original magnification, × 400). (B) Quantitation of mean TUNEL-positive cells/field. are expressed as mean ± SD. *P < 0.01 compared with control; ^#^*P *< 0.05, ^##^*P *< 0.01, compared with MCD feeding mice; ^$^*P *< 0.01, compared with MCD+Ad-GFP treated mice. Slides are representative of 6 animals per group.

As shown in Figure 1, TUNEL-positive cells appeared occasionally in the liver sections of control mice, but were frequently observed in mice fed with the MCD diet. TUNEL-positive cells were decreased by hemin or Ad-HO-1 administration. Treatment with hemin plus Ad-HO-1 did not further reduce the number of apoptotic cell. In contrast, TUNEL-positive cells were markedly increased by receiving ZnPP-IX compared with the MCD diet alone.

### Effect of HO-1 induction on hepatic oxidative stress

**Figure 2:**
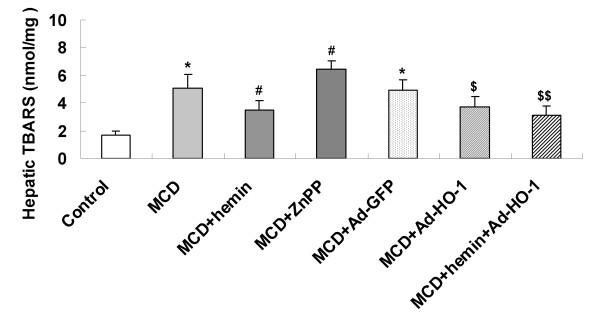
**Effects of the MCD diet and treatment with hemin and/or Ad-HO-1 or ZnPP-IX on hepatic lipoperoxide content measured as thiobarbituric acid-reactive substrances (TBARS)**. Data are expressed as mean ± SD (n = 6 per group). *P < 0.01, **P < 0.001, compared with control; ^#^*P *< 0.05, ^##^*P *< 0.01, compared with MCD feeding mice; ^$^*P *< 0.05, ^$$^*P *< 0.01, compared with MCD+ Ad-GFP treated mice.

Hepatic level of oxidative stress was analyzed by TBARS assay (Figure 2). Mice fed with MCD diet resulted in a prominent increase in TBARS level compared with that of the control mice. A significant reduction of TBARS contents was noted after hemin treatment for 4 weeks compared to MCD-treated mice. A similar effect was observed by Ad-HO-1 gene transfer compared to mice administered Ad-GFP. The combination of hemin and Ad-HO-1 failed to show an additive effect on suppressing TBARS levels. However, the level of TBARS were markedly increased in mice treated with ZnPP-IX than those fed MCD diet only (Figure 2). Measurement of hepatic TBARS revealed that HO-1 induction protected mice from oxidative injury.

### Induction of HO-1 by hemin and/or Ad-HO-1

**Figure 3:**
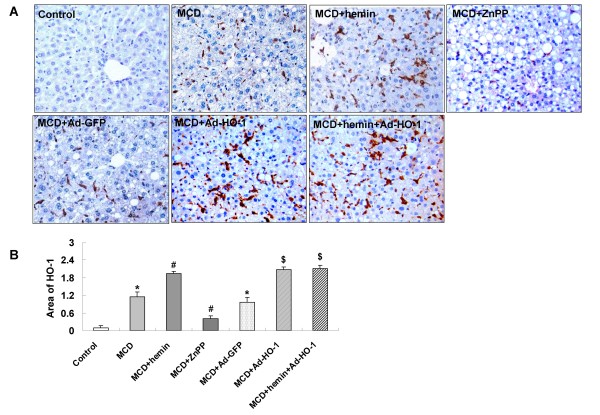
**Effects of hemin and/or Ad-HO-1 on hepatic HO-1 protein expression in the liver of mice**. (A) Immunostaining for HO-1 protein. (B) Effect of hemin and/or Ad-HO-1 on quantitative protein expression of HO-1. The expression of HO-1 was estimated by average area density (areas of positive cells/total areas) (original magnification, × 200). Data are expressed as the mean ± SD (n = 6 per group). *P < 0.001, compared with control; ^#^*P *< 0.01, compared with MCD feeding mice; ^$^*P *< 0.01, compared with MCD+ Ad-GFP treated mice.

Immunohistochemical staining for HO-1 was barely detectable in mice fed control diet (Figure 3), HO-1 staining was increased in liver sections of steatohepatitis mice fed the MCD diet, and appeared to be mainly in hepatocytes and kupffer cells both in the nuclei and cytoplasm, whereas in the sections of MCD-fed mice treated with hemin or Ad-HO-1, strong and dense HO-1 immunoreactivity was observed, which paralleled the improvement in histological severity of steatohepatitis. Co-administration of hemin and Ad-HO-1 had no better effect on up-regulation of HO-1 expression. Conversely, in ZnPP-IX treatment mice, hepatic protein expression of HO-1 was not observed in company with a pronounced liver injury (Figure 3).

### Effect of HO-1 on the expression of CYP2E1 and Cyt-c

**Figure 4:**
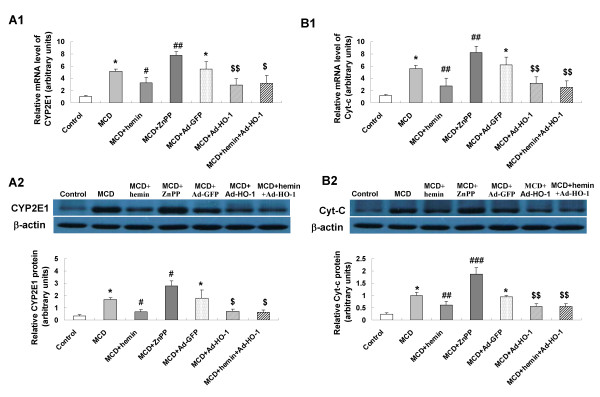
**Effects of HO-1 on hepatic expression of cytochrome p450 2E1 (CYP2E1) (A) and cytochrome c (Cyt-c) (B)**. mRNA expression of CYP2E1 (A1) and Cyt-c mRNA(B1) was examined by real-time quantitative PCR; and protein expression of CYP2E1 (A2) and Cyt-c (B2) were measured by Western blot. Data are expressed as the mean ± SD (n = 6 per group). *P < 0.001, compared with control mice; ^#^*P *< 0.05, ^##^*P *< 0.01, ^###^*P *< 0.001, compared with MCD mice; ^$^*P *< 0.05, ^$$^*P *< 0.01, compared with MCD+ Ad-GFP treated mice.

The mRNA and protein expressions of lipid peroxidation mediator CYP2E1 were induced by MCD diet (Figure 4A1 and 4A2). The release of Cyt-c resulted from mitochondrial dysfunction also highly enhanced in MCD diet mice (Figure 4B1 and 4B2). Treatment with hemin or Ad-HO-1 prevented CYP2E1 induction and Cyt-c release. A similar effect was observed in hemin plus Ad-HO-1 group. Conversely, hepatic mRNA and protein expression of CYP2E1 and Cyt-c was further up-regulated by ZnPP-IX treatment as compared to MCD group.

### Effect of HO-1 induction on the expression of genes related to apoptosis

**Figure 5:**
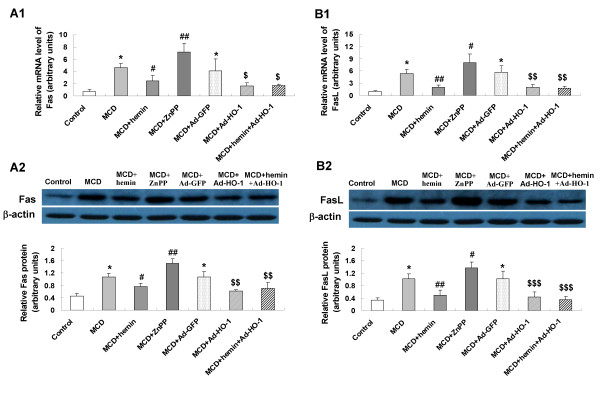
**Effects of HO-1 on expression of FAS and FASL in the liver of mice**. mRNA expression of Fas (A1) and Fasligand (FasL) (B1) was examined by real-time PCR; protein expression of Fas (A2) and FasL (B2) were measured by Western blot. Data are expressed as the mean ± SD (n = 6 per group). *P < 0.001, compared with control mice; ^#^*P *< 0.05, ^##^*P *< 0.01, compared with MCD mice; ^$^*P *< 0.05, ^$$^*P *< 0.01, ^$$$^*P *< 0.01, compared with MCD+ Ad-GFP treated mice.

**Figure 6:**
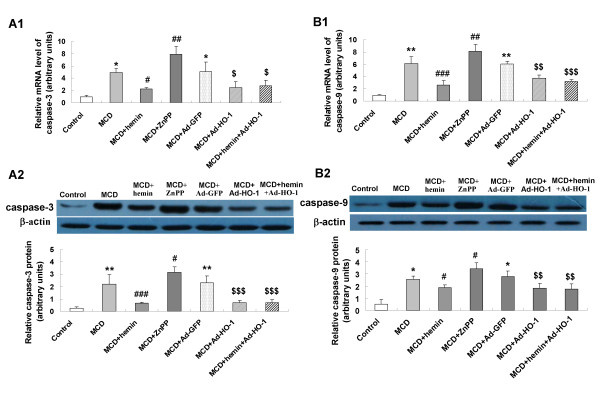
**Effects of HO-1 on expression of caspase 3 and casepase 9 in the liver of mice**. mRNA expression of casepase 3 (A1) and casepase 9 (B1) was examined by real-time PCR; protein expression of casepase 3 (A2) and casepase 9 (B2) were measured by Western blot. Data are expressed as the mean ± SD (n = 6 per group). *P < 0.01, ** *P *< 0.001 compared with control mice; ^#^*P *< 0.05, ^##^*P *< 0.01, ^###^*P *< 0.01, compared with MCD mice; ^$^*P *< 0.05, ^$$^*P *< 0.01, ^$$$^*P *< 0.01, compared with MCD+ Ad-GFP treated mice.

**Figure 7:**
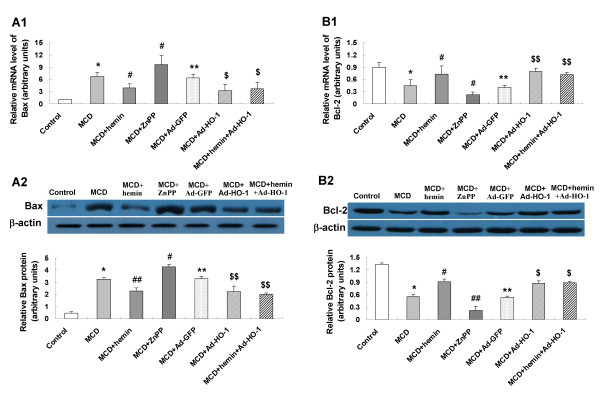
**Effects of HO-1 on expression of Bax and Bcl-2 in the liver of mice**. mRNA expression of Bax (A1) and Bcl-2 (B1) was examined by real-time PCR; protein expression of Bax (A2) and Bcl-2 (B2) were measured by Western blot. Data are expressed as the mean ± SD (n = 6 per group). *P < 0.01, ** *P *< 0.001 compared with control mice; ^#^*P *< 0.01, ^##^*P *< 0.01, compared with MCD mice; ^$^*P *< 0.05, ^$$^*P *< 0.01, compared with MCD+ Ad-GFP treated mice.

To seek the role of HO-1 induction on cell apoptosis in the pathogenesis of steatohepatitis, we investigated expression levels of the key apoptosis-related genes. In MCD feeding mice, mRNA and protein expression of Fas (Figure 5A), FasL (Figure 5B), caspase-3 (Figure 6A), caspase-9 (Figure 6B), Bax (Figure 7A) had a marked elevation and the anti-apoptosis gene Bcl-2 (Figure 7B) was dramatically decreased. The expression of Fas/FasL, caspase-3, caspase-9 and Bax were down-regulated and Bcl-2 was up-regulated by hemin or Ad-HO-1 administration. No further effect on regulating apoptosis genes expression was observed in administration of hemin and Ad-HO-1. In contrast, the expression of Fas/FasL, caspase-3, caspase-9 and Bax were further up-regulated and Bcl-2 was further down-regulated by ZnPP-IX compared with mice fed a MCD diet.

## Discussion

Following the MCD diet for 4 weeks, mice rapidly and consistently developed a steatohepatitis with steatosis, mixed inflammatory cell infiltration and hepatocellular necrosis which is in line with our previous report [20] and histologic similarities to human disease [21]. Accompany with the histological changes, there was a marked induction of hepatocyte apoptosis compared with the control mice. Treatment with HO-1 selective inducer hemin or Ad-HO-1 significantly attenuated the MCD-induced hepatic apoptotic injury by induction of hepatic HO-1 protein levels, which was evidenced by reduction of hepatocyte apoptosis and amelioration of liver histology. However, the combination of Ad-HO-1 and hemin did not show a synergetic effect. In contrast, a pronounced liver injury and lowered HO-1 immunostaining was presented by giving ZnPP-IX, a specific competitive inhibitor of HO-1 [22]. It was reported that human liver generated comparable amounts of the HO-derived products in both kupffer cells and hepatocytes, and HO-1 induction protected against ischemia/reperfusion injury, oxidative stress, inflammation, apoptosis, transplant rejection, and other conditions [12]. Our data show that HO-1 is up-regulated by hemin or Ad-HO-1, which might play an important role in protection against hepatic apoptotic injury caused by MCD diet.

In fatty liver, surplus of fatty acids and excessive oxidation lead to production of reactive oxygen species (ROS) and oxidative stress, which will trigger inflammatory response and apoptosis [23]. Excessive production of ROS generated from microsomal, mitochondrial, and other pro-oxidant pathways may eventually overwhelm antioxidant defenses and generate highly toxic lipid peroxides. The increased release of lipid peroxidation products bind to mitochondrial proteins, promote cytochrome c release [24], and then contribute to cell death. CYP2E1, a microsomal fatty acid oxidizing enzyme, is known to be a significant source of ROS [25]. Ethanol and dietary, as well as endogenously produced fatty acids, are inducers of and substrates for CYP2E1 [26]. It generates high amounts of hydrogen peroxide in the presence of oxidizable cosubstrates [27]. Enhanced CYP2E1 induction could oxidize mitochondrial DNA, proteins and lipids, and trigger hepatic tumor necrosis factor-alpha (TNF-α) formation by activating nuclear factor-kappaB (NF-κB), thus further increase mitochondrial ROS formation, and then lead to the inflammatory recruitment and apoptosis from oxidative stress [23]. In the present study, we found enhanced oxidative stress in the MCD-diet mice. Lipid peroxidation, as reflected by hepatic TBARS concentration and the mRNA and protein expression of CYP2E1 were significantly increased in mice fed a MCD diet compared with control mice. Treatment with hemin or Ad-HO-1 reduced hepatic TBARS levels and CYP2E1 expression. Consistent with our findings, Zhu et al. [28] have demonstrated that HO-1 up-regulation increased resistance to oxidant-mediated cytotoxicity and reduced basal prooxidant levels. These results suggest that up-regulation of HO-1 expression attenuate oxidative stress and inhibit progression of liver injury, which probably is due to alleviated lipid peroxidation and CYP2E1 reduction.

It is now recognized that increased hepatocyte apoptosis is a prominent feature in steatohepatitis and correlates strongly with clinical and histologic disease severity [29,30]. The association between increased oxidative stress and a high rate of cellular apoptosis has been reported in hepatocytes [31]. To elucidate the mechanisms by which apoptosis occurs in the liver may provide an insight into the pathogenesis of steatohepatitis and identify possible treatments. We demonstrated that oxidative stress related hepatocyte apoptosis was enhanced by feeding mice MCD diet. Treatment with hemin or Ad-HO-1 resulted in significantly resistance to apoptosis, evidenced by diminution of the TUNEL-positive cells and down-regulated mRNA and protein expressions of key pro-apoptotic factors Fas/FasL, caspase-3, caspase-9, Bax and Cyt-c, and markedly increased anti-apoptotic Bcl-2. Overproduction of ROS might induce apoptosis by inducing FasL to interact with Fas and formed a death-inducing signal complex (DISC) [32], activated caspase cascade including caspase-8 and caspase-3 to recruit hepatocytes to apoptosis [33]. Also ROS might promote onset of the mitochondrial permeability transition (MPT) by inducing translocation of Bax from cytosol to mitochondria and lead Cyt-c to release [34] to cause the cell apoptosis by formation of a complex with apoptotic protease-activating factor-1 and activation of caspase-9 and its downstream effectors caspases-3, 6 and 7 [35,36]. Thus, a common feature of the death receptor- and mitochondrion-dependent apoptosis is the activation of caspase-3 [37]. It was suggested that HO-1 mediated protection was accompanied by significantly reduced caspase-3 activation [38]. HO-1 regulated mitochondrial transport carriers and function by activating Bcl-2 and Bcl-xL, preventing Cyt-c release and activation of caspases [39]. Increased HO-1 expression increased Bad, inhibited Cyt-c release and increased cell survival [40,41]. Collectively, the protective effect of HO-1 on oxidative damage-induced apoptosis may be mediated via both the extrinsic pathway and the intrinsic apoptosis signaling pathways. HO-1 provides both antioxidant and anti-apoptotic properties maybe due to its products of BV/BR, iron and CO. BV/BR has been shown to defense against reactive oxygen species [38] and BV adjuvant therapy has been shown to protect rat liver grafts from ischemia/reperfusion injury through suppressing Cyt-c release [42]. Iron induces ferritin, which in turn prevents lipid peroxidation [43]. CO has been exhibited anti-inflammatory and anti-apoptotic properties, which are thought to be mediated by activation of p38 mitogen-activated protein kinase (p38 MAPK) signaling pathway [44-46]. It has also been reported that the anti-apoptotic effect of CO involves in inhibition of Fas/FasL expression, and other apoptosis-related factors including caspases (especially caspase-3), mitochondrial Cyt-c release, and poly adp-ribose polymerase cleavage [47]. Clearly, further studies are still necessary to clarify both how and at which level HO-1 affects the two apoptosis signaling pathways.

In summary, the present study suggest that induction of HO-1 by pretreatment with hemin or Ad-HO-1 attenuated hepatic apoptosis injury in MCD diet-fed mice, which maybe regarded to alleviation of lipid peroxidation, suppression of CYP2E1 expression, down-regulation of Fas/FasL, caspase-9 and caspase-3 expression, reduction of Cyt-c release and modification of Bcl-2/Bax ratio. The present study provided evidences for the protective role of HO-1 in preventing nutritional steatohepatitis through suppressing hepatocyte apoptosis in mice.

## List of Abbreviations

HO-1: heme oxygenase-1; MCD: methionine-choline deficient; NASH: non-alcoholic steatohepatitis; ZNPP-IX: zinc protoporphyrin IX; TUNEL: terminal deoxynucleotidyl transferase dUTP nick-end labeling; ROS: reactive oxygen species; CYT-C: cytochrome c; CYP2E1: cytochrome p4502E1; TBARS: thiobarbituric acid reactive substances.

## Competing interests

The authors report no conflicts of interest. The authors alone are responsible for the content and writing of the paper.

## Authors' contributions

YN and JY designed the research; RW, SZ, FH, WW, LK and NF performed the research; YN and RW analyzed data; YN, RW and JY wrote the paper. All authors read and approved the final manuscript.
